# Interface polarization model for a 2-dimensional electron gas at the BaSnO_3_/LaInO_3_ interface

**DOI:** 10.1038/s41598-019-52772-8

**Published:** 2019-11-07

**Authors:** Young Mo Kim, T. Markurt, Youjung Kim, M. Zupancic, Juyeon Shin, M. Albrecht, Kookrin Char

**Affiliations:** 1 Institute of Applied Physics, Dept. of Physics and Astronomy, Seoul National University, Seoul, 08826 Korea; 2 Leibniz-Institut für Kristallzüchtung, Berlin, 12489 Germany

**Keywords:** Electronic properties and materials, Semiconductors, Surfaces, interfaces and thin films, Electronic properties and materials, Semiconductors, Surfaces, interfaces and thin films

## Abstract

In order to explain the experimental sheet carrier density *n*_2D_ at the interface of BaSnO_3_/LaInO_3_, we consider a model that is based on the presence of interface polarization in LaInO_3_ which extends over 2 pseudocubic unit cells from the interface and eventually disappears in the next 2 unit cells. Considering such interface polarization in calculations based on 1D Poisson-Schrödinger equations, we consistently explain the dependence of the sheet carrier density of BaSnO_3_/LaInO_3_ heterinterfaces on the thickness of the LaInO_3_ layer and the La doping of the BaSnO_3_ layer. Our model is supported by a quantitative analysis of atomic position obtained from high resolution transmission electron microscopy which evidences suppression of the octahedral tilt and a vertical lattice expansion in LaInO_3_ over 2–3 pseudocubic unit cells at the coherently strained interface.

## Introduction

Two-dimensional electron gases (2DEGs) that reside at the interface of semiconductor heterostructures have made significant contribution to advancement of science as well as to field effect device applications, for example high electron mobility transistors (HEMT)^1,2^. Forming 2DEG requires a confining potential and a source of electrons that populate this channel. The archetype of a 2DEG is realized by modulation doping^1,3^, a way of remote doping, in GaAs/AlGaAs heterostructures, where the confining potential is achieved by the band bending at the interface. The system has enabled 2DEGs with very high mobility, which in turn led to the quantum Hall effect^4,5^. In wide bandgap semiconductors such as GaN/AlGaN^6–8^ and ZnO/MgZnO interfaces^9–12^ that exhibit wurtzite structures, the confinement of electrons at the interface is facilitated by the polarization discontinuity at the interface, given by the difference in spontaneous and piezoelectric polarization due to epitaxial strain. In a perovskite heterostructure, 2DEG-like behavior with superconductivity at the SrTiO_3_/LaAlO_3_ polar interface has been studied extensively^13–16^. The origin of the 2DEG at the SrTiO_3_/LaAlO_3_ interface is attributed in literature to the charge discontinuity^13,14^ and the charge transfer^17^ between the wider bandgap polar perovskite LaAlO_3_, i.e. a material with an alternating sequence of layers with opposite non-zero ionic charge (LaO^+^ and AlO_2_^−^), and the non-polar SrTiO_3_ consisting of non-charged SrO° and TiO_2_° layers. The charge transfer across the interface would lead to a carrier density of ½ electron per interface unit cell area, amounting to 3.2 × 10^14^ cm^−2^ ^17^. However, experimental proofs of this model remain particularly challenging due to the difficulty in controlling the unintentional doping of SrTiO_3_ by oxygen vacancies^15,16^.

Recently, heterostructures formed of the orthorhombic LaInO_3_ (LIO) and cubic BaSnO_3_ (BSO) have been attracting considerable attention^18–20^. BSO has a bandgap of 3.1 eV and, in contrast to SrTiO_3_, possesses high electron mobility (~300 cm^2^/Vs) at room temperature and at high carrier concentration (~10^20^ cm^−3^)^21,22^. The excellent chemical stability of BSO including its oxygen stoichiometry presents an opportunity to create and deliberately manipulate the 2DEG channel at the interface of BSO without considering oxygen vacancies as intrinsic donors. LIO is of an orthorhombic GdFeO_3_ structure and its lattice constant matches very well with that of BSO; its pseudocubic lattice constants (*a*_pc_), calculated from the three lattice constants (a = 5.9404 Å, b = 5.7229 Å, and c = 8.2158 Å) in the orthorhombic structure^23^, is *a*_pc_ = ${(\sqrt{{a}^{2}+{b}^{2}}/2\cdot c/2)}^{1/2}$ = 4.117 Å. Considering the 4.116 Å lattice constant of cubic BSO, the growth of LIO on BSO is coherent. Depositing such BSO/LIO heterostructures on both SrTiO_3_ and MgO substrates^18,20^, high mobility (~100 cm^2^/Vs) field effect transistors with large I_on_/I_off_ (~10^9^) were demonstrated.

Based on the experimental results showing an enhancement of the interface conductance at the BSO/LIO interface with increasing LIO thickness within the first 4 pseudocubic monolayers of growth, we come up with an interface polarization model in which a large polarization decays after the first 2 pseudocubic unit cells of LIO and eventually disappears after the next 2 unit cells. Entering this model into 1-dimensional (1D) Poisson-Schrödinger equation, we predict a 2DEG at the interface of BSO/LIO that is confined within a 2 nm thick layer. Based on this model, we are able to explain the entire set of experimental data, namely the observed dependence of our interface conductance on both the thickness of the LIO layer and the La doping of the BSO layer. Quantitative high resolution transmission electron microscopy evidences a gradual change of the octahedral tilt from that of the cubic BSO to that of the orthorhombic LIO together with a localized increase of the vertical lattice parameter within the first 2–3 pseudocubic unit cells near the BSO/LIO interface. We associate this structural modification at the interface with the presence of an interface polarization at the BSO/LIO heterointerface.

## Results and Discussion

**Figure 1:**
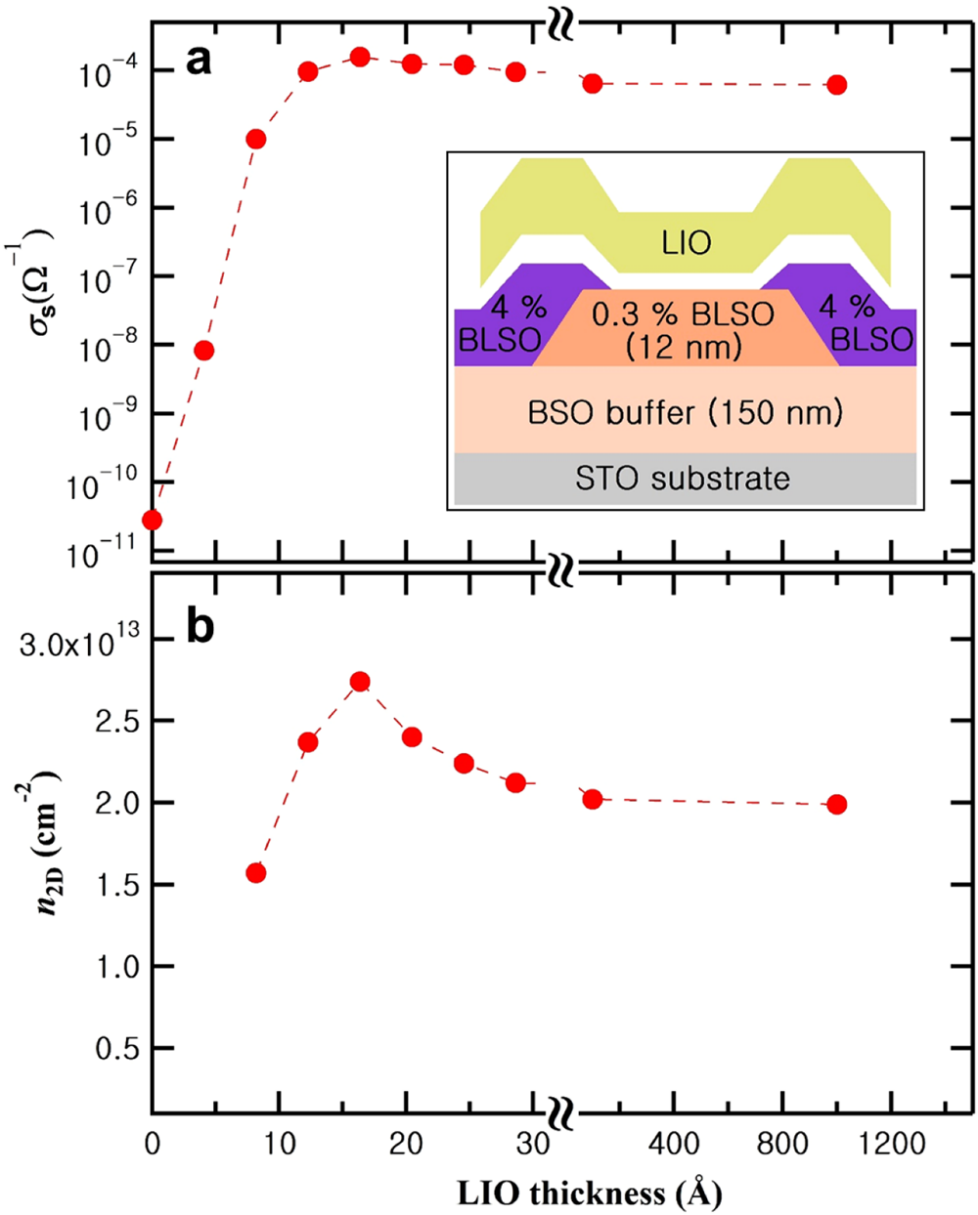
Electrical transport properties of the 0.3% BLSO/LIO interface at room temperature. (**a**) Conductance (*σ*_s_) as a function of the LIO thickness, (**b**) sheet carrier density (*n*_2D_) as a function of the LIO thickness. The inset is the schematic cross-sectional diagram of the processed device structure.

Figure 1 shows the measured electrical properties of the BSO/LIO interface, as a function of the thickness of the LIO layer on top of a 12 nm thick 0.3% (*N*_d_ = 4.3 × 10^19^ cm^−3^) La-doped BSO (BLSO). It is important to note that despite of the doping with La as high as 0.3%, the BLSO layer itself remains insulating (>10^10^ Ω). In our previous work^24,25^ we found that this is due to the large concentration of deep acceptor states (4~6 × 10^19^ cm^−3^, i.e. overcompensating the La donors) resulting from the high density of threading dislocations (~10^11^ cm^−2^) and cation vacancies in BSO films. As soon as the first unit cell of LIO is deposited, we observe a sudden enhancement in conductance. Unlike the SrTiO_3_/LaAlO_3_ system^14^ we do not see a critical thickness of LIO for 2DEG behavior. The conductance continuously increases until it reaches its maximum at a LIO thickness of 4 unit cells (~16 Å), followed by a slow decrease as the LIO layer becomes thicker. The observed enhancement of conductance with increasing LIO thickness has been reproduced in several batches of BSO/LIO heterostructures^19,20^. The sheet carrier density (*n*_2D_) obtained by Hall effect measurements, shown in Fig. 1(b), follows the same trend, i.e. *n*_2D_ increases until a LIO thickness of 4 unit cells and then decreases slowly.

A way to calculate both the charge distribution and the band bending in a consistent manner over an entire semiconductor heterostructure is the Poisson-Schrödinger formalism. It calculates the final potential landscape relative to the Fermi level, leading to *n*_3D_ and *n*_2D_ along with the confinement length. For this we used the self-consistent 1D Poisson-Schrödinger band calculator provided by Snider^26^. In our simulations, we have used ohmic boundary conditions in which the Fermi levels are in the middle of the bandgaps at the two end boundaries. The important input parameters for the simulation are as follows.

(i) Material parameters like the effective masses, the dielectric constants of BSO and LIO, and the conduction band offset between BSO and LIO are known from previous studies^18,21,27^ and are summarized in Table 1.

**Table 1:** Materials parameters used for LIO and BSO.

Materials	*E*_g_ (eV)	Δ*E*_*C*_ to BSO (eV)	ε_r_	${m}_{e}^{\ast }/{m}_{0}$	*E*_D_ (eV)	*E*_A_ (eV)
LIO	5.0	1.6	38	0.46	2.5	—
BSO	3.1	—	20	0.42	−0.63	1.55
(ii) The band alignment relative to the Fermi level on the BSO side of the heterostructure can be well controlled by intentional doping of BSO with by La doping (*N*_d_ = 4.3 × 10^19^ cm^−3^ for 0.3% La-doped BSO). However, as mentioned before, BSO films grown on foreign substrates have a high density of threading dislocations (~10^11^ cm^−2^) acting as electron traps (due to the presence of cation dangling bonds at the threading dislocation core). We introduce deep acceptor states in BSO to model such traps, in which the ionization energy of the deep acceptors is set to be half of the band gap. Based on our previous results the deep acceptor densities (*N*_DA_) are set to be 4 × 10^19^ cm^−3^ for films grown on MgO substrates^24^ and 6 × 10^19^ cm^−3^ on SrTiO_3_ substrates. For computational efficiency, acceptors are set only in regions where there are non-negligible electron densities, for example as shown in Fig. S1.
(iii) The concentration of unintentional deep level dopants in LIO will affect the band alignment relative to the Fermi level on the LIO side of the BSO/LIO heterointerface. Their role on the band alignment at the BSO/LIO interface and thus the sheet carrier concentration of the 2DEG channel will be discussed later.
(iv) Furthermore, the polarization inside LIO in order to accommodate the polarization discontinuity between the nonpolar BSO and the polar LIO is an important parameter which influences the band alignment. In our calculations we considered a uniform polarization inside the whole LIO layer as well as a polarization localized near the BSO/LIO interface.

**Figure 2:**
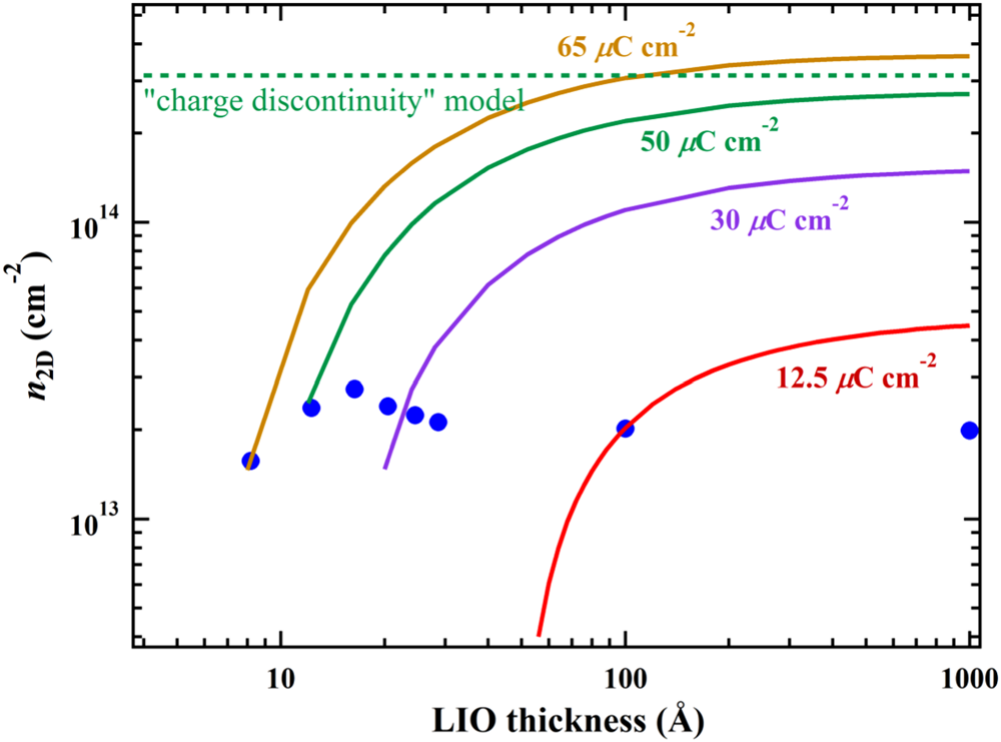
The calculated sheet carrier density (*n*_2D_) as a function of the LIO thickness with various uniform polarization values inside LIO. For P = 50 μC/cm^2^, the polarization value of the alternating LaO^+^ and InO_2_^−^ layers, *n*_2D_ approaches the predicted value *n*_2D_ = 3 × 10^14^ cm^−2^ by the “charge discontinuity” model in the thick LIO limit.

At an ideal boundary, where the polarization is uniform and extended infinitely in the direction perpendicular to the boundary, a polarization discontinuity ΔP across the boundary will result in the surface charge density *n*_2D_ = ΔP/e. Figure 2 shows the *n*_2D_ calculated as a function of LIO thickness with various uniform polarization P inside LIO. First of all, it is important to note that, when P = 50 μC/cm^2^, the expected value for alternating LaO^+^ and InO_2_^−^ layers, the sheet carrier density approaches in the thick film limit the value of *n*_2D_ = P/e = 3.1 × 10^14^ cm^−2^ predicted by the “charge discontinuity” model. It is also clear that no value of uniform polarization reproduces for the entire LIO layer thickness range the experimental data set shown in Fig. 1(b). In order to fit the first data point at LIO = 8 Å, a high polarization value of 65 μC/cm^2^ is needed. However, to fit the *n*_2D_ values for larger thickness of the LIO layer, a much lower polarization is needed. Therefore, we were forced to consider a highly non-uniform polarization, where the high polarization rapidly diminishes after the first 2 unit cells of LIO.

**Figure 3:**
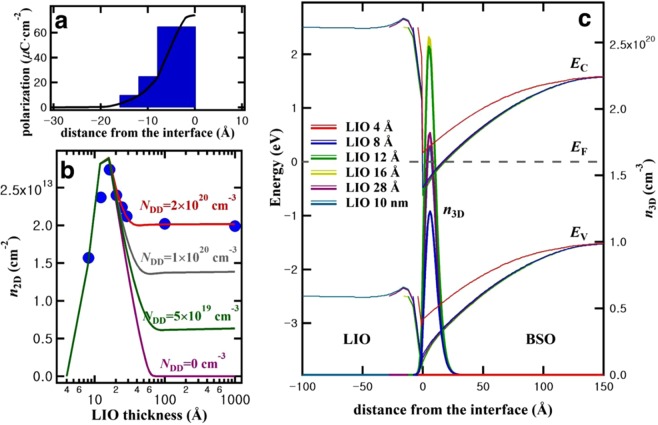
Self-consistent Poisson-Schrödinger simulation for BLSO(0.3%)/LIO interface. (**a**) A model for interface polarization used for simulation. The curve is a guide for eyes. (**b**) Comparison of *n*_2D_ from experiments (dots) with those by simulation (lines) for various *N*_DD_. (**c**) Band diagrams and *n*_3D_ near the interface. Deep donor density *N*_DD_ = 2 × 10^20^ cm^−3^ inside LIO is assumed.

To test the idea whether such interface polarization results in a confinement which in turn enables the formation of a 2DEG, we simulated the BLSO/LIO heterostructure with an LIO layer showing a polarization decaying after two unit cells of LIO at the interface. Based on the results in Fig. 1 in which the interface conductance peaks for 4 unit cell thick LIO layer, we assume an interface polarization in LIO which disappears after 4 unit cell thickness. As shown in Fig. 3(a), the best fit to the experimental data as function of LIO film thickness in Fig. 3(b) was obtained if the polarization in the first two pseudocubic unit cells is set to be 65 μC/cm^2^, the third unit cell to be 25 μC/cm^2^, and the fourth and the last unit cell to be 10 μC/cm^2^. It has to be mentioned here that these polarization values critically depend on the considered conduction band offset. The value of 1.6 eV used here is deduced from a tunneling experiment which tends to underestimate the barrier height^18,28^. If, for example, a conduction band offset of 1.9 eV is used instead of 1.6 eV, a polarization of 50 μC/cm^2^ in the first 2 unit cells generates almost the same *n*_2D_.

An example of a sample structure used in our calculation with detailed materials parameters for each layer is presented in Fig. S1 in the case of a 10 nm thick LIO film. Using such a model with interface polarization, the calculated band diagrams and electron concentrations of the BLSO(0.3%)/LIO interface as dependent on the LIO thickness are shown in Fig. 3(c). The results of the calculation can be summarized as following. The interface polarization is tilting the bands in the LIO layer which together with the ohmic boundary condition leads to an overall downward bending of the bands at the BSO/LIO interface. This in turn creates an electron confinement channel in BSO. With increasing thickness of LIO up to 4 unit cells (the thickness of the layer that possess polarization) the confinement becomes deeper and the electron concentration in the confined channel therefore increases. Our model generates a 2DEG state in BSO with the confinement length of about 2 nm. This length scale is consistent with the recent 3-dimensional and 2-dimensional thermoelectric measurement of BSO epitaxial thin films^29^. In addition such a 2DEG band structure may be confirmed by a thickness and element sensitive measurement technique such as x-ray photoelectron spectroscopy.

The calculated sheet carrier density *n*_2D_ matches well with the experimental data of Fig. 1, when the LIO film thickness is less than 4 unit cells, as shown in Fig. 3(b). However, in the simulation, the sheet carrier density *n*_2D_ decreases rapidly for films thicker than 4 unit cells due to the lack of polarization after 4 unit cells and eventually *n*_2D_ disappears when LIO becomes 7 nm thick. This is due to the negatively charged surface when the polarization ends at the 4 unit cells and the lack of any available charges to screen it. In order to mitigate the rapid decrease of *n*_2D_, we introduced deep donor states in LIO. These deep donor states, for example oxygen vacancies in LIO, don’t ionize spontaneously at room temperature but can be ionized to become positively charged when the Fermi level is lower than the intrinsic Fermi level, resulting in screening the negatively charged surface. This is well illustrated in Fig. 3(b), where the deep donor states in LIO effectively screen the potential of the interface polarization and allow the LIO band to be flat when LIO becomes thicker. We also found that the deep donor states in the flat band region do not affect the band diagram and the *n*_2D_; the deep donor states within 5 nm from the interface suffice. A deep donor density (*N*_DD_) of 2 × 10^20^ cm^−3^ in LIO, which, for example, corresponds to LaInO_2.993_ near the interface, was found to fit the experimental sheet carrier density *n*_2D_ for LIO films thicker than 4 unit cells, as shown in Fig. 3(b). Experimentally we have seen no change in conductance after oxygen annealing, suggesting that the oxygen vacancies in LIO, if they are indeed the deep donors in our model, are stable since they may be related with the strain and/or the microstructure of LIO. In our model there are 2 sources for the electrons in the quantum well: one from the conduction band of BSO and the other from the LIO side. In the BSO side, the Fermi level close to the conduction band minimum suggests that excited electrons near the interface can move to the quantum well. In the LIO side, near the interface (0 < LIO thickness < 1.6 nm) the donor electrons can tunnel into the quantum well. Additionally in the region where the Fermi level becomes lower than the deep donor (1.6 nm < LIO thickness < 3 nm), the deep donor states should become empty, suggesting that the deep donor states are ionized and the resulting donor electrons will end up where the conduction band is lower than the Fermi level, namely in the quantum well.

**Figure 4:**
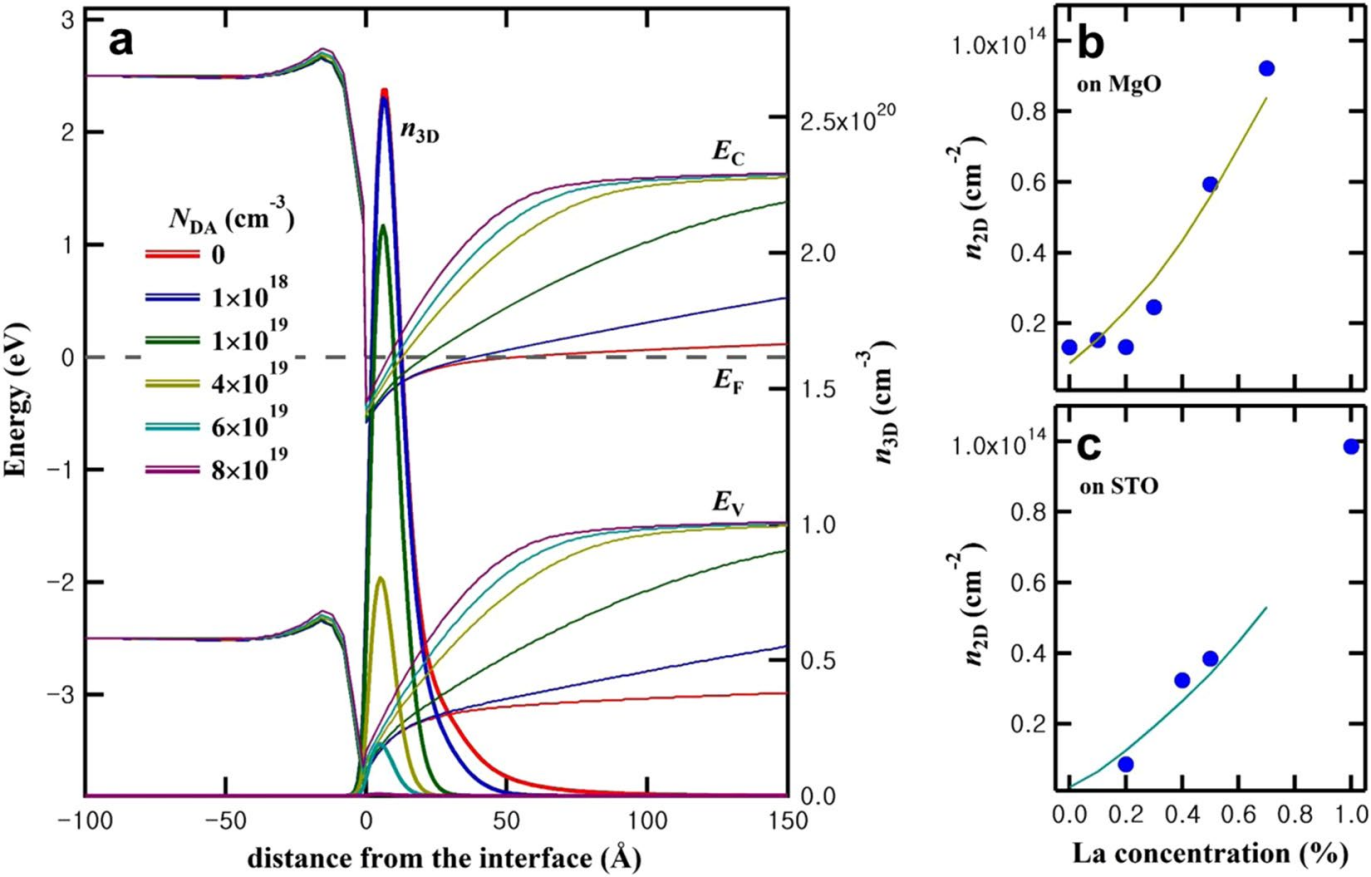
Calculations for *N*_DD_ = 2 × 10^20^ cm^−3^ in 10 nm LIO. (**a**) Dependence of band diagrams and *n*_3D_ on the deep acceptor density (*N*_DA_) without La doping (*N*_d_ = 0). (**b**) Comparison of the donor density (*N*_d_) dependence of *n*_2D_ by simulation (line) with those from experiments (dots) on MgO substrates. (*N*_DA_ = 4 × 10^19^ cm^−3^) (**c**) Comparison of the donor density (*N*_d_) dependence of *n*_2D_ by simulation (line) with those from experiments (dots) on SrTiO_3_ substrates. (*N*_DA_ = 6 × 10^19^ cm^−3^).

**Figure 5:**
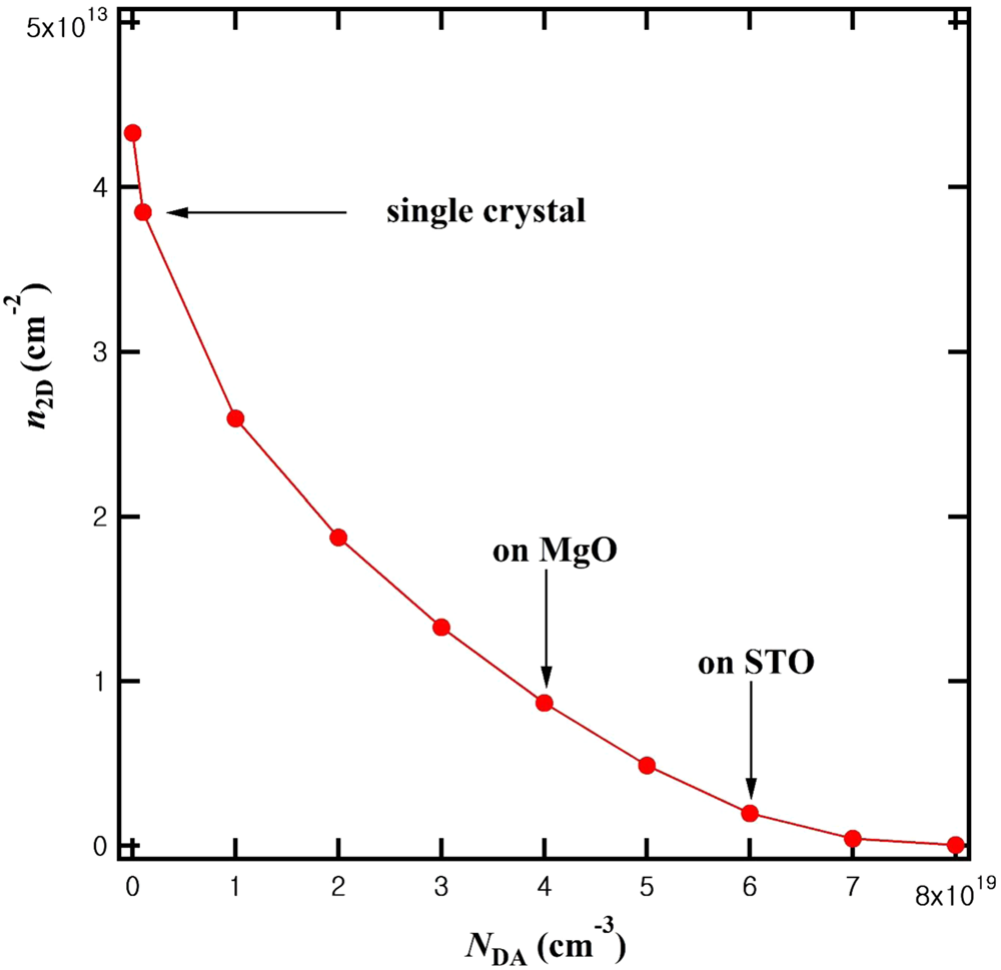
*n*_2D_ of LIO(10 nm)/BSO(undoped) as a function of *N*_DA_ in BSO.

Switching to the BSO side, to see the influence of deep acceptor states in BSO, we simulated the BSO(undoped)/LIO interface as a function of the deep acceptor density (*N*_DA_). As is evident from Fig. 4, the deep acceptors in BSO, in addition to their role as electron traps, screen the electric field inside BSO and thus reduce the width and to a small extent also the depth of the confined channel. As *N*_DA_ increases, the bands of BSO change faster near the interface and become flat after screening of the electric field by the ionized deep acceptors. In case of growth on MgO substrates the simulation matches well with the experimental results^24^ for a deep acceptor density of 4 × 10^19^ cm^−3^, as shown in Fig. 4(b). In particular, the *n*_2D_ ~ 1 × 10^13^ cm^−2^ without La doping of BSO on MgO substrates can be explained. In case of growth on SrTiO_3_ substrates^19^, it was found that BSO(undoped)/LIO didn’t show noticeable conductance enhancement and the magnitude of conductance enhancement for doped BLSO/LIO was smaller than that for the MgO substrate case^20^. Setting for heterostructures grown on SrTiO_3_ substrates the deep acceptor density to be 6 × 10^19^ cm^−3^, we found that the experimental data were well fitted by the simulation as shown in Fig. 4(c). Our simulation shows that the difference of BSO films on MgO and SrTiO_3_ substrates can be well modeled by varying the deep acceptor densities. This is consistent with the idea in our previous report^20^ that the BSO films on SrTiO_3_ substrates have a larger deep acceptor density (more cation dangling bonds in the threading dislocation cores). More importantly, our simulation also predicts that in case the deep acceptor density in BSO becomes less than 10^18^ cm^−3^, as is expected for example for single crystals with a much lower dislocation density, the *n*_2D_ will be about 4.0 × 10^13^ cm^−2^ without any La doping in BSO, as shown in Fig. 5. This value agrees with the maximum *n*_2D_ we found earlier^19^ as a function of La doping on SrTiO_3_ substrates.

**Figure 6:**
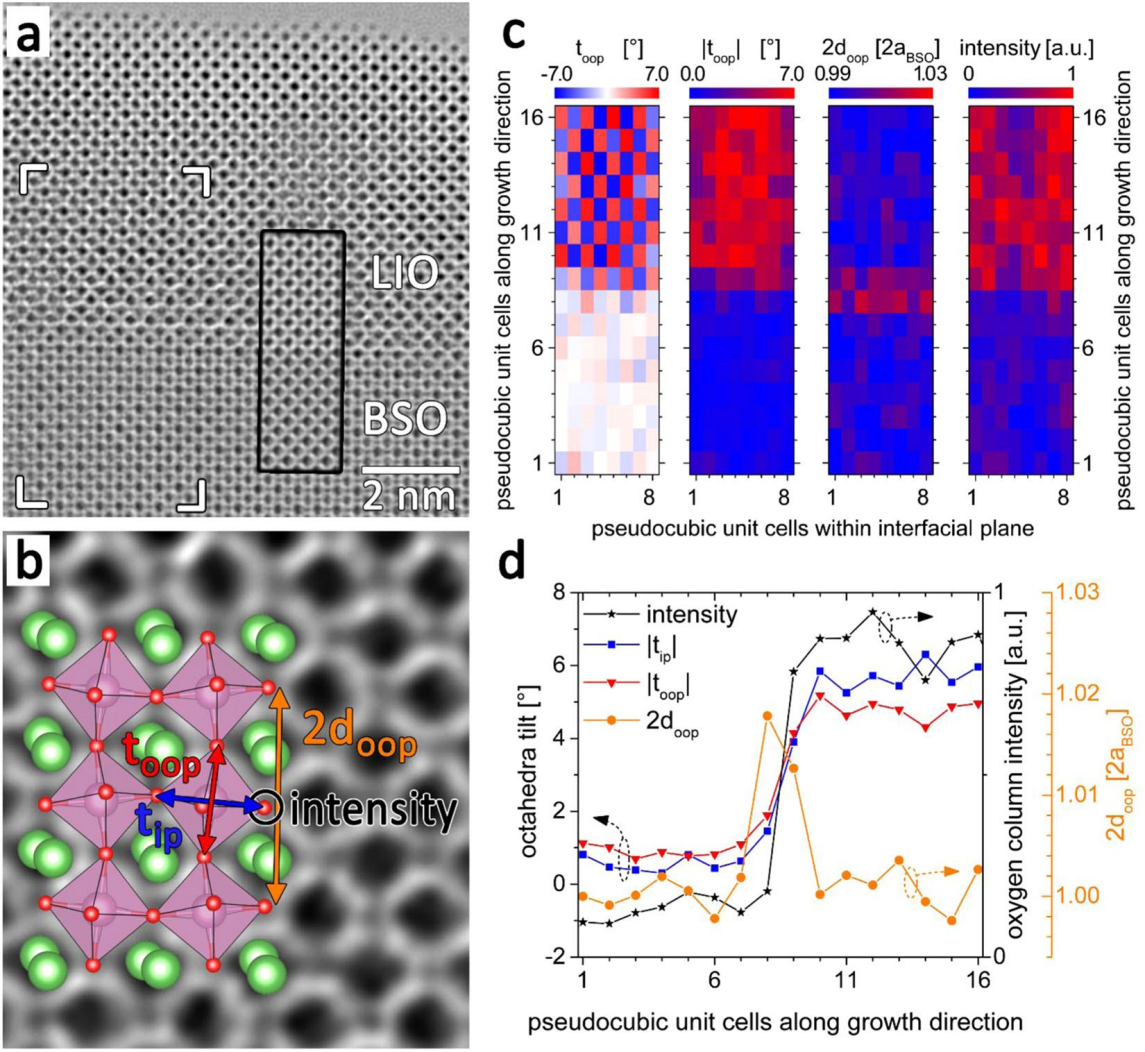
(**a**) Cross-sectional HRTEM image of the BSO/LIO interface. The inset with the black frame is a HRTEM image simulation. (**b**) Magnified view of LIO from (**a**) together with a stick-and-ball-model of the orthorhombic crystal structure of LIO^23^ indicating the tilt of the oxygen octahedra (violet). Green, violet and red balls represent La, In and O atomic columns, respectively. The tilt angle of the out-of-plane and in-plane oxygen octahedra axis (t_oop_ and t_ip_), the out-of-plane lattice parameter (2d_oop_), and the intensity of the oxygen atomic column of the corresponding octahedra, respectively, are indicated in the image. (**c**) Color coded maps evaluated from the region indicated by the white frame in (**a**), from left to right: tilt angle t_oop_, its modulus |t_oop_|, the out-of-plane lattice parameter 2d_oop_, and the intensity of the oxygen atomic column of the corresponding octahedra. (**d**) Line profiles across the BSO/LIO interface of the laterally averaged values of |t_oop_|, |t_ip_|, 2d_oop_, and the intensity of oxygen atomic columns.

The remaining question is whether and what kind of structural modification at the BSO/LIO heterostructure can support the idea of interface polarization hypothesized from our Poisson-Schrödinger calculations. To answer this question, we investigated the atomistic structure of the interface by aberration corrected high resolution transmission electron microscopy (HRTEM). Figure 6(a) shows the amplitude of an exit wave reconstruction retrieved from a cross-sectional HRTEM defocus series of the BSO/LIO interface together with an image simulation (inset with the black frame). Atomic columns appear dark in the image. Having a closer look at the intensity of the whole HRTEM pattern and at individual atomic columns reveals that although the differences in atomic numbers between BSO and LIO is small (ΔZ between Ba-La and Sn-In is only 1!), a distinction is possible. This is confirmed by comparing the experimental image with a simulation which reproduced the slightly lower intensity as well as the lower overall image contrast in the BSO layer. Analyzing the HRTEM pattern shows that the LIO film grows coherently on the BSO layer. However, the film consists of approximately 5~20 nm wide domains which correspond, due to the reduced symmetry of the orthorhombic lattice of LIO, to the different 90° rotated variants of the pseudocubic unit cell. Only for domains with the orthorhombic c-axis along the viewing direction the tilt of oxygen octahedra can be analyzed from the HRTEM image because only for these domains the octahedra are tilted along the beam direction with the same sense (see Fig. 6(b)). In this case oxygen atoms at the corners of the octahedra are aligned as straight columns along the electron beam direction and their positions and spacing can be measured in the HRTEM image. Using this procedure we analyzed the tilt angle of the in-plane (t_ip_) and out-of-plane axis (t_oop_) of oxygen octahedra, their respective modulus (|t_ip_| and | t_oop_|), the out-of-plane lattice parameter (2d_oop_), and the intensity of oxygen atomic columns of the corresponding oxygen octahedra. These quantities are schematically indicated in Fig. 6(b) and color coded representations of them measured in a region across the BSO/LIO interface marked by the white frame in the HRTEM image in Fig. 6(a) are shown as maps in Fig. 6(c). Line scans of the laterally averaged values of the aforementioned quantities are shown in Fig. 6(d). Looking first at the intensity of oxygen columns we can clearly identify the BSO/LIO interface from chemistry point of view by the abrupt change in intensity. The BSO layer extends from layer 1–8 along the growth direction in Fig. 6(c,d), LIO from layer 9–16. Let us now turn to the determined tilt angle of the out-of-plane axis of individual oxygen octahedra of the perovskite network across the BSO/LIO interface (t_oop_, left map in Fig. 6(c)). In the BSO layer the predominant white color indicates zero tilt of the octahedra with a standard deviation of the tilt measurement of 1.0°. In the LIO film adjacent columns of oxygen octahedra, both along the in-plane as well as the out-of-plane direction (i.e. the growth direction), are tilted oppositely leading to the overall red-blue checkerboard pattern in the left map in Fig. 6(c). The same behavior was found for the tilt angle of the in-plane axis of oxygen octahedra (t_ip_, not shown here). The modulus of the out-of-plane tilt angle |t_oop_| is displayed in the second map from left in Fig. 6(c). In the BSO layer, the modulus of the octahedra tilt does not exceed our measurement precision of 1°. On the other side, in the LIO film the modulus of the octahedra tilt amounts to a constant value of approximately 5.5°. The most interesting observation is the behavior at the interface between the BSO layer and the LIO film. The increase of the modulus of the octahedra tilt from BSO to LIO occurs gradually over a distance of 2–3 pseudocubic perovskite layers (see red and blue graphs in Fig. 6(d)). At the same time we observe for the same layers at the BSO/LIO interface that exhibit a suppression of octahedra tilt an increase of the out-of-plane lattice parameter by more than 1% (see second map from right in Fig. 6(c) and orange graph in (d)) although the pseudocubic lattice parameters of BSO and LIO differ by less than 0.01 Å (corresponding to ≈ 0.2% difference). The accumulated displacement of the LIO perovskite lattice with respect to that of BSO due to the increased lattice parameter at the interface amounts to approximately 0.13 Å.

Such gradual suppression of octahedral tilt has been observed for the BiFeO_3_/(LaSr)MnO_3_ interface^30^, where the Fe-O-Fe bond angle was modified over a distance of 10 unit cells by 6° near the interface. Recently a similar suppression of octahedral tilt and its polar effect has been reported for the SrTiO_3_/LaAlO_3_ interface^31,32^. It is generally believed that the octahedral tilt is antiferrodistortive, as opposed to ferroelectric distortions caused by a relative shift of cations with respect to anions^33^. Therefore it is not surprising that its suppression by coherent epitaxial growth on a cubic lattice can create the interface polarization. However, the quantitative relation between the structural modifications observed here and the interface polarization is still lacking. It will also not be surprising that the interface polarization state for a BSO/LIO heterostructure may be completely different from the polarization state in LaAlO_3_/SrTiO_3_, given that the LaAlO_3_ layer grown coherently on SrTiO_3_ is under a large 2.5% tensile strain unlike our case of almost perfect lattice constant matching between BSO and LIO. Furthermore, there are 3 different orientations of domains of orthorhombic LIO when grown on cubic BSO. More atomic resolution structural study along with theoretical calculations will be needed to fully answer the relation between the epitaxial strain and the interface polarization. Given the fact that for the present case the coherent epitaxial growth leads to structural modifications at the BSO/LIO interface and the resulting interface polarization, which in turn enables the formation of a 2DEG state, one may be able create polarization and interface conduction even for a non-polar perovskite interface by exploiting large epitaxial strain, for example as in the SrTiO_3_/CaZrO_3_ case^34^.

### Summary

In this paper we employ a combined experimental and theoretical approach to study the conductance enhancement at the non-polar/polar perovskite BSO/LIO interface. The basis of our investigation is a set of experimental data of the sheet carrier density *n*_2D_ of BSO/LIO heterostructures obtained by a systematic variation of the LIO film thickness, BSO doping concentration and defect density reproduced in several batches of samples. Using self-consistent 1D Poisson-Schrödinger band calculation we found that only a model based on interface polarization which quickly diminishes after the first 2 unit cells of LIO reproduces the entire set of experimental data of the thickness, doping, and substrate dependent variation of the sheet carrier density *n*_2D_. The calculations also predict a 2DEG state in BSO with a confinement length of 2 nm. The assumption of our model of a localized polarization present only near the BSO/LIO interface is supported by aberration corrected HRTEM which provides evidence of structural modifications on the same length scale of 2–3 pseudocubic unit cells as the hypothesized interface polarization, namely a gradual suppression of oxygen octahedral tilt together with a localized increase of the lattice parameter in the polar orthorhombic LIO near the interface, due to its coherent epitaxial growth on the cubic BSO. The 2DEG state at the BSO/LIO polar interface may have high density (*n*_2D_ ~ 10^14^ cm^−2^, *n*_3D_ ~ 10^21^ cm^−3^) with a potentially large Rashba-type spin-orbit coupling energy.

### Methods

We used the same geometric patterns as the ones employed in our previous papers on BSO/LIO interface^19,20^. The vertical structure of the interface is shown in the inset of Fig. 1. Samples were grown on TiO_2_-terminated SrTiO_3_ (001) substrates at 750 °C in 100 mTorr of oxygen pressure by pulsed laser deposition using KrF excimer laser with energy fluence in the range of 1.2~1.5 J/cm^2^. All targets were provided by Toshima Manufacturing Co. in Japan. We first deposited a 150 nm undoped BSO buffer layer to reduce the density of threading dislocations. Secondly, 12 nm of 0.3% (*N*_d_ = 4.3 × 10^19^ cm^−3^) La-doped BSO (BLSO) square channel layer was grown and 4% BLSO contact layers were deposited on the four corners of the channel. Lastly, LIO layer was deposited on the channel layer. This way we can measure the conductance before and after the interface formation. A Keithley 4200 SCS was used to measure electrical properties of channels. In order to obtain *n*_2D_, Hall effect measurements were conducted when channels were sufficiently conductive, using Van der Pauw geometry of a 2 mm size square.

High resolution transmission electron microscopy (HR-TEM) measurements were performed with an aberration corrected FEI Titan 80–300 operated at 300 kV. Specimens for TEM investigations were prepared by tripod polish and Ar ion milling at liquid nitrogen temperature using a Gatan PIPS at sequentially reducing ion beam energies between 4.0 ~ 0.2 keV. Because of the orthorhombic symmetry of LIO we had to measure two pseudocubic spacings as a representation of the out-of-plane lattice parameter. A single pseudocubic spacing measurement is modulated by a checkerboard like modulation of smaller and larger spacings which prevents a clear visualization of the present strain. Since we have measured the double pseudocubic lattice spacing as a representation of the out-of-plane spacing, the spatial resolution of our strain measurement, i.e. the information from where the increased lattice spacing originates, is limited to approximately 2 pseudocubic/perovskite unit cells. Therefore we have quantified the accumulated displacement instead of a local strain value.

## Supplementary information

Supplementary informaton
