# Supplementary material for: Interface polarization model for a 2-dimensional electron gas at the BaSnO3/LaInO3 interface
Source: Sci Rep. 2019 Nov 7;9:16202. doi: 10.1038/s41598-019-52772-8 (PMC6838460; doi:10.1038/s41598-019-52772-8)
Supplement: Supplementary file 1 — Supplementary informaton [file 41598_2019_52772_MOESM1_ESM.docx]

Supplementary Information for

Interface polarization model for a 2-dimensional electron gas

at the BaSnO_3_/LaInO_3_ interface

Young Mo Kim^†^, T. Markurt^†^, Youjung Kim, M. Zupancic, Juyeon Shin,

M. Albrecht, and Kookrin Char^*^

^†^These authors contributed equally to this work.

^*^Correspondence to: kchar@phya.snu.ac.kr


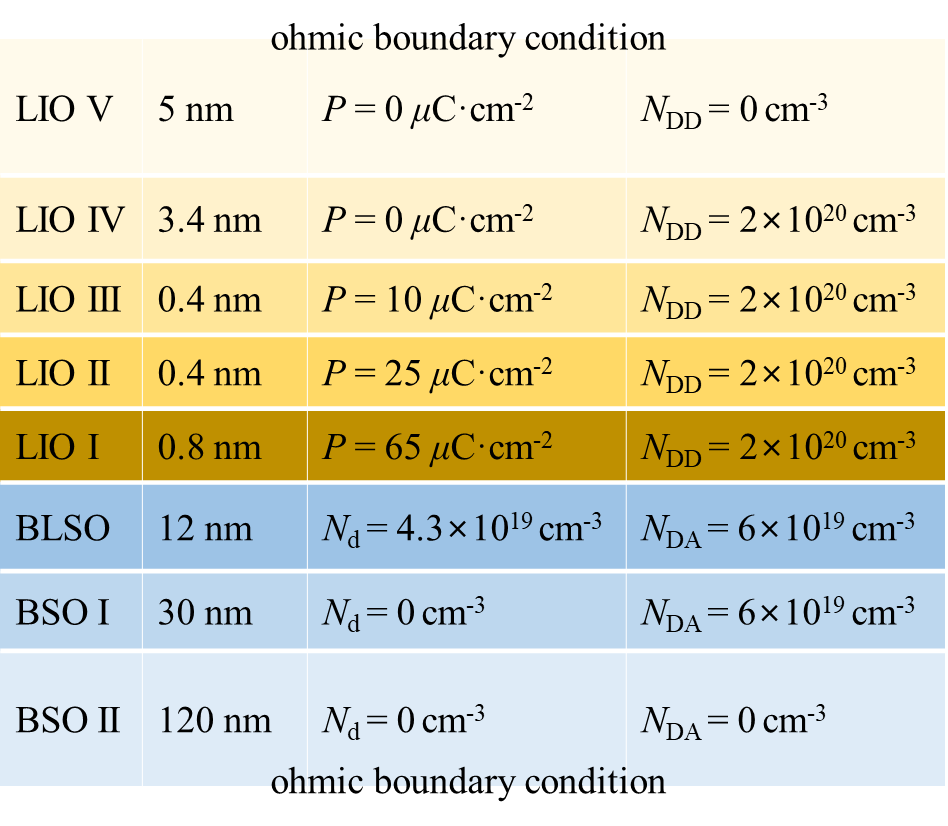


Fig. S1. Parameters in each layer for LIO(10 nm)/0.3 % BLSO(12 nm)/BSO(150 nm) on SrTiO_3_ simulation using an “interface polarization” model. Note that *N*_d_ < *N*_DA_ in 0.3 % BLSO, rendering the BLSO layer insulating even with 0.3 % La doping
